# High Urinary Sodium Concentrations in Severe SIADH: Case Reports of 2 Patients and Literature Review

**DOI:** 10.3389/fmed.2022.897940

**Published:** 2022-05-04

**Authors:** Lynette Mei Yi Lee, Sarah Ying Tse Tan, Wann Jia Loh

**Affiliations:** ^1^Department of Endocrinology, Sengkang General Hospital, Singapore, Singapore; ^2^Department of Endocrinology, Changi General Hospital, Singapore, Singapore

**Keywords:** hyponatremia, syndrome of inappropriate antidiuretic hormone (SIADH), urinary sodium, traumatic brain injury, cerebral salt wasting (CSW), case report, SIADH literature review

## Abstract

We present two cases of severe hyponatremia secondary to syndrome of inappropriate secretion of antidiuretic hormone (SIADH) with very high urine sodium concentrations (>130 mmol/L). The first patient had hyponatremia from traumatic brain injury (TBI) while the second case had a history of recurrent SIADH triggered by various causes including gastritis. In both cases, fluid administration and/or consumption worsened the hyponatremia. Although a low urine sodium of <30 mmol/L is highly suggestive of hypovolemic hyponatremia and good response to saline infusion, there is lack of clarity of the threshold of which high urine sodium concentration can differentiate various causes of natriuresis such as SIADH, renal or cerebral salt wasting. Apart from high urine osmolality (>500 mOsm/kg), persistence of high urine sodium concentrations may be useful to predict poor response to fluid restriction in SIADH. More studies are needed to delineate treatment pathways of patients with very high urine osmolality and urine sodium concentrations in SIADH.

## Introduction

Hyponatremia affects 20–30% of hospital admissions (1, 2) with the syndrome of inappropriate secretion of antidiuretic hormone (SIADH) being a prominent cause of hyponatremia that is common and yet challenging to manage. Hyponatremia of all severity is associated with increased mortality; with up to 33% mortality reported in severe hyponatremia (3).

SIADH is defined as the presence of hypo-osmolality and high urine osmolality in the euvolemic state with no evidence of renal salt wasting in an individual with normal renal, adrenal, thyroid, cardiac, and liver function (4, 5). It may be precipitated by central nervous system disorders, neoplasia, pulmonary disorders, pain, and medications (6). Traumatic brain injury (TBI) causes hyponatremia, often due to SIADH or cerebral salt wasting (CSW) (7, 8). Assessing volume status is a key step in establishing the etiology of hyponatremia, however, it is subjective and generally inaccurate, except in severely hypervolemic or hypovolemic cases (5, 9). Assessment of urine osmolality and urine sodium concentration is more reliable in aiding clinicians to appropriately classify the hyponatremia to major etiological causes (5, 9). Urine sodium >30 mmol/L in a euvolemic patient suggests SIADH, in the absence of diuretics, hypothyroidism and adrenal insufficiency.

We present two unusual cases of severe hyponatremia secondary to SIADH, one due to traumatic brain injury and another due to underlying gastritis causing dyspepsia. In both instances, urine sodium concentrations were very high (>130 mmol/L). We also performed a literature review of reported cases of SIADH with high urine sodium concentrations.

## Case 1

**Figure 1:**
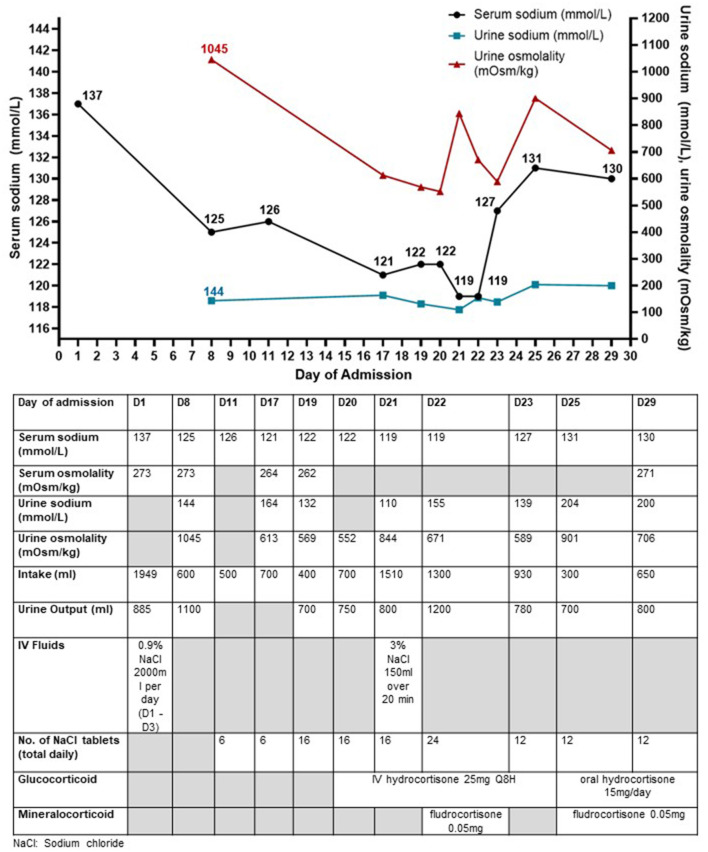
Trend of serum sodium concentrations, urine sodium concentrations, and urine osmolality along with the interventions taken in Case 1 who presented with traumatic brain injury and later diagnosed with SIADH. The urine biochemistry was performed on urinary spot samples. The urine sodium concentrations were persistently raised ≥130 mmol/L throughout the hospital stay except on day 21.

A previously well 40-year-old Indonesian male presented with transient loss of consciousness following a fall-from-standing height while working as a sailor aboard a ship. He sustained traumatic brain injury (TBI) with subarachnoid and subdural hemorrhages and multiple facial fractures with raised serum creatine kinase 1014 U/L (NR <210U/L). His initial Glasgow coma scale score was 14. His injuries were managed conservatively. His initial serum sodium concentration was 140 mmol/L with normal renal panel and serum potassium 3.8 mmol/L. Full blood count and liver function test were unremarkable. Intravenous (IV) 0.9% sodium chloride (NaCl) infusion 2L/day was given for the first 3 days of admission in view of the significantly raised creatine kinase worrisome for early-onset rhabdomyolysis. However, serum sodium progressively fell to 125 mmol/L, and he was referred to the Endocrinology service on day 8 of admission (D8). His vital signs were stable with no hypotension or tachycardia (heart rate 81 beats/min, blood pressure 133/87 mmHg). He was clinically euvolemic with moist mucous membranes, clear lung fields on auscultation, and no peripheral oedema. Investigations revealed hypo-osmolar hyponatremia with markedly elevated urine sodium (144 mmol/L) and urine osmolality (1,045 mOsm/kg). His renal and thyroid function were normal and peak serum cortisol was normal (611 nmol/L) after a 1mcg ACTH-stimulation test. The clinical impression was SIADH from TBI. Intravenous sodium chloride drip was stopped, and he was restricted to 750 ml of fluid per day and given additional sodium chloride tablets (up to 272 mmol/day). Urine sodium concentration was persistently high even after saline infusion drip was discontinued (Figure 1).

Despite strict fluid restriction, his serum sodium continued to downtrend to 121 mmol/L (Figure 1). Urine sodium concentration (132–164 mmol/L) and urine osmolality (569–613 mOsm/kg) remained significantly raised. Differential diagnoses for the etiology of his refractory hyponatremia included (a) mild central hypocortisolism post-TBI, (b) SIADH, and (c) CSW. Although initial peak cortisol response to 1-mcg ACTH stimulation test exceeded 500 nmol/L with normal ACTH levels, this did not rule out acute central hypocortisolism precipitated by the recent TBI due to preserved adrenal tissue to external ACTH stimulation during acute pituitary injury (10). Thus, an insulin tolerance test was performed to assess the hypothalamic-pituitary-adrenal axis. He received 0.35 u/kg of regular insulin and achieved symptomatic hypoglycemia (plasma glucose 2.4 mmol/L) with a peak cortisol level of 502 nmol/L. The test was stopped prior to achieving the target of plasma glucose <2.2 mmol/L because the patient could not tolerate the hypoglycemia symptoms. In view of borderline cortisol response and the declining sodium concentrations, a trial of IV hydrocortisone replacement was started, but this did not result in an improvement of hyponatremia. The hyponatremia did not fulfill the criteria for CSW because he was clinically euvolemic with normal serum urea and creatinine and he did not have polyuria. Intravenous fluid administration worsened the hyponatremia. Fractional excretion of uric acid (FeUA) performed on D22 of admission was low at 9.29% (<11%) when patient was euvolemic was suggestive although not specific for SIADH (11). A repeat FeUA on D27 admission was still low at 7.98%.

Despite fluid restriction, high load of sodium chloride tablets and hydrocortisone, serum sodium fell further to 119 mmol/L with persistently raised urine sodium (110 mmol/L) and urine osmolality (844 mOsm/kg) and the patient reported worsening vertiginous giddiness associated with nausea. His renal function remained normal without potassium or magnesium derangement throughout the admission. In view of symptomatic hyponatremia, he was given 150 ml of IV 3% NaCl which transiently raised serum sodium level to 122 mmol/L. However, serum sodium level decreased to 119 mmol/L the next day (D22). Sodium chloride tablets were increased to 24 tablets daily and a decision was made for trial of oral fludrocortisone 0.05 mg (D22). This led to significant improvement in serum sodium to 127 mmol/L with a mild decrease in urine sodium from 155 to 139 mmol/L (D23) although the rise may not be attributed solely to fludrocortisone, in view of the relatively minor decrease in urine sodium concentration, and the subsequent rise in urine sodium concentration (200 mmol/L on D29). The usage of sodium chloride tablets and persistence of fluid restriction also contributed to the improvement in hyponatremia. Oral fludrocortisone was served every other day (0.05 mg) with gradual improvement in serum sodium (131 mmol/L on D25). After low dose fludrocortisone was started, there was also successful tapering of oral sodium chloride tablets down to 12 tablets a day, with further tapering till it was stopped 6 weeks later. He was discharged well on D29. A week later at the Endocrine clinic, his serum sodium normalized (138 mmol/L) while on lower doses of sodium chloride tablets, low dose hydrocortisone, and fludrocortisone 0.05 mg every other day. Fludrocortisone and hydrocortisone were stopped. His sodium level remained normal (142 mmol/L) without any medications 6 weeks after discharge. During this prolonged hospital stay for hyponatremia, the patient in case 1 expressed his concern that the symptomatic dizziness from hyponatremia would persist and affect his ability to work. He was relieved that both his symptoms and hyponatremia resolved completely. He was able to resume work subsequently.

## Case 2

A 53-year-old Indian man with well-controlled type 2 diabetes and hypertension presented with 1 week of severe persistent abdominal pain, bloating, and poor appetite. He was admitted to the hospital under the Gastroenterology department for gastritis and treated with IV proton pump inhibitors (PPI) and analgesia including tramadol. His serum sodium level was 127 mmol/L on admission and was started on IV 0.9% NaCl 1L/day for presumptive hypovolemic hyponatremia. However, serum sodium dropped to 117 mmol/L on day 3 of admission (D3), and he was urgently referred to the Endocrinology service for management of hyponatremia.

Further history revealed that although he was eating poorly due to abdominal discomfort, he had increased his water intake to 2–3L/day to relieve his bloating. He was nauseous but did not have vomiting or diarrhea. He was alert and clinically stable on examination (blood pressure 121/76, heart rate 73 bpm, GCS 15). He was clinically euvolemic. He had hypo-osmolar hyponatremia with markedly elevated urine sodium concentration (187 mmol/L) and urine osmolality (494 mOsm/kg). Serum creatinine and urea levels were not raised, thyroid hormone levels were normal, and peak serum cortisol after a 250 mcg ACTH stimulation test was normal at 653 nmol/L. His HbA1c was 7.1%. Serum pro-brain natriuretic peptide level was normal at <50 pg/ml. Thus, a diagnosis of SIADH was made, likely triggered by pain. Tramadol was stopped on D3 due to association with SIADH, however he continued to have worsening hyponatremia.

On review of his medical records, he had previous severe symptomatic hyponatremia (serum sodium concentration of 110 mmol/L) 4 years ago, necessitating admission to the intensive care unit and treatment with hypertonic saline. On further questioning, he recalled that he had developed dizziness and poor appetite several days prior to admission but was still drinking large volumes of water up to 3 L/day because he was concerned about dehydration after moderate-intensity exercise. Symptoms worsened and culminated in him vomiting and losing consciousness, which then led to the hospital admission. Laboratory tests then similarly showed markedly elevated urine sodium (176 mmol/L) and urine osmolality (600 mOsm/kg), in keeping with SIADH. His subsequent outpatient serum sodium levels performed at the general practice were normal until current admission.

**Figure 2:**
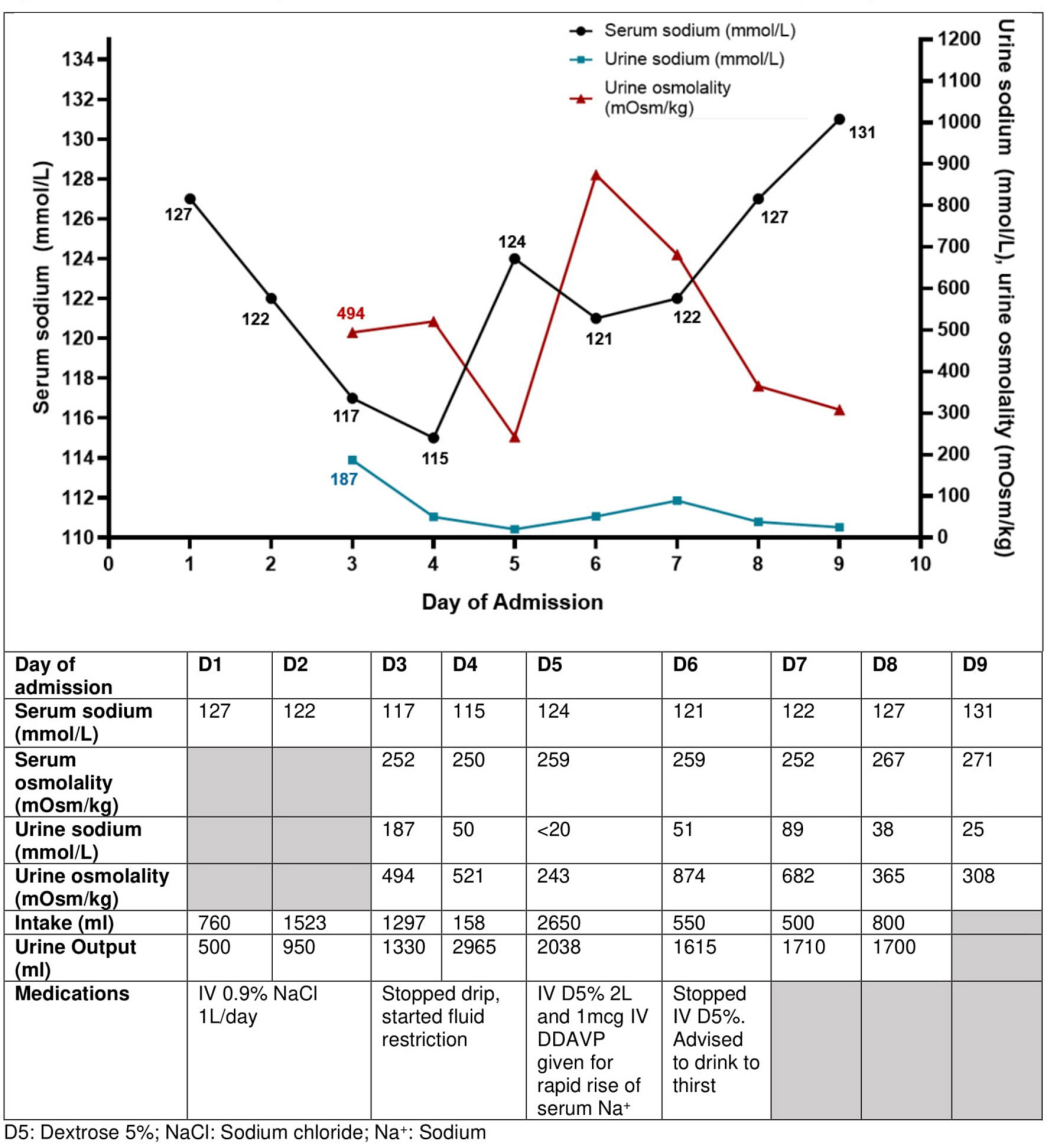
Trend of serum sodium concentrations, urine sodium concentrations, and urine osmolality with the interventions taken in Case 2 with recurrent SIADH. The current SIADH episode illustrated here was caused by dyspepsia from gastritis. His urine sodium concentration was elevated at 187 mmol/L initially and reduced as his hyponatremia due to SIADH improved. The urine biochemistry was performed on urinary spot samples.

During this current admission, in view of the diagnosis of SIADH, IV fluids were stopped, and fluid restriction was started. Further investigations were performed for the abdominal pain and SIADH including a computed tomography scan of the abdomen, magnetic resonance imaging of the brain, and a chest radiograph, all of which did not show any abnormalities. His symptoms improved with proton pump inhibitor therapy and his appetite gradually improved. This was associated with a rise in serum sodium level, a fall in urine sodium levels, and a rise in urine output, indicative of free water excretion (day 4 admission [D4], Figure 2), which led the serum sodium levels to rise rapidly from 115 mmol/L to 124 mmol/L in 24 h (D5, Figure 2). He was given IV dextrose infusion and 1 dose of desmopressin to dampen the rapid rise in sodium level, and serum sodium was successfully re-lowered to 121 mmol/L. The ensuing rise in sodium was appropriate and corresponded with a gradual fall in urine sodium and osmolality levels (Figure 2). Esophagogastroduodenoscopy showed mild antral gastritis with *Helicobacter. Pylori* detected, for which antibiotics were given for eradication of bacteria. The recurrent severe SIADH episodes that led to prolonged hospital stays were frustrating for this patient. He was appreciative of the advice to avoid such episodes by avoiding excessive water intake especially when a stimulus of SIADH was present, along with the explanation of the physiology of water-sodium balance in a patient prone to SIADH.

## Discussion

In this series of 2 cases, we reported that urine sodium concentration can be very high (>130 mmol/L) in the presence of SIADH. Interestingly, despite similar degree of nadir hyponatremia, high urine sodium concentrations (150–200 mmol/L) and raised urine osmolality of at least 490 mOsm/kg, the severity of the etiology of the SIADH were on opposite ends of the spectrum; the first case was a severe TBI causing impaired consciousness, while the other case was a relatively benign disorder of dyspepsia due to gastritis. Neither were on diuretics nor had renal injuries to account for the excessive urine sodium loss. The onset of hyponatremia was precipitated by excessive fluid administration (case 1) or consumption (case 2) in the setting of SIADH. Fluid restriction was undertaken in both cases, but the hyponatremia in case 1 was refractory to fluid restriction and required additional therapeutics including fludrocortisone, while the hyponatremia in case 2 responded too rapidly necessitating the use of desmopressin to dampen the rise of serum sodium level. Through our literature review (Table 1), we found that other groups have reported moderate-severe hyponatremia cases secondary to SIADH with urine sodium concentrations >100 mmol/L suggesting that although uncommon, a very high urine sodium concentration does not make the diagnosis of SIADH less likely but rather is a marker of severity of SIADH (12).

**Table 1:** Case reports of SIADH with high urinary sodium (>100mmol/L).

**Case**	**Initial serum sodium (mmol/L)**	**Urinary sodium (mmol/L)**	**Urinary osmolality (mOsm/kg)**	**Reason why not CSW**	**Etiology of SIADH**	**Treatment of SIADH**	**Outcome**
Amoako et al. (13)	118	150	NA	No head injury	Duloxetine and paralytic ileus	Cessation of duloxetine	Good recovery
Andersen et al. (14)	124	135	548	No head injury	Pneumonia	Tolvaptan	Death from multi-organ failure
Chang et al. (15)	122	159	710	No hypotension or polyuria	Fall with subarachnoid hemorrhage	Fluid restriction Hypertonic saline	Good recovery
Chua et al. (16)	131	222	742	Hyponatraemia worsened with saline challenge	Severe head injury with skull fractures	Hypertonic saline and fluid restriction	Good recovery
Dick et al. (17)	117	112	920	Euvolemic, improved with fluid restriction	Traumatic brain injury	Fluid restriction Demeclocycline	Good recovery
Huda et al. (18)	122	141	896	Euvolemic	Left cerebral hemisphere stroke	Demeclocycline Tolvaptan Hypertonic saline	Right hemiparesis due to stroke
Kumar et al. (19)	119	132	203	Responded to fluid restriction	Traumatic brain injury	Fluid restriction	Good recovery
Van der Voort et al. (20)	114	283	880	Responded to fluid restriction	Traumatic brain injury	Fluid restriction Oral salt and urea loading	Good recovery

One of the factors contributing to the dilemma in diagnosing SIADH accurately is the assessment of “euvolemia” (9). This is largely dependent on the subjective clinical assessment of hydration status, which is generally not very accurate. A study showed that inexperienced doctors using algorithms incorporating urine osmolality and sodium concentration with less reliance on volume status assessment had better diagnostic accuracy than senior physicians not using algorithms (21). Thus, the European guidelines recommended the use of urine osmolality and urine sodium concentrations as key steps in the assessment of the etiology of hyponatremia (9). Another reason contributing to the diagnostic dilemma is that SIADH is a diagnosis of exclusion (4, 9, 22) and the first-line therapy of SIADH (fluid restriction) does not always work (23), perplexing the physician further. While urine sodium concentrations <30 mmol/L signify a low effective arterial blood volume and have a high negative predictive value for SIADH (5), it is unclear what the upper threshold is to differentiate SIADH from other causes of natriuresis (e.g., renal salt wasting or CSW). The cases presented here illustrate the diagnostic challenge of SIADH that physicians face, where in both cases, intravenous fluid administration was attempted as the initial step of therapy. An unusual feature was the markedly raised urine sodium concentrations leading to investigations for other causes of hyponatremia, such as CSW (case 1) and renal abnormalities (case 2), leading to the delay in the diagnosis and treatment of SIADH. Although the high urine sodium concentrations may have been increased by the intravenous saline infusion the day before, the urine sodium concentrations were found to be similarly raised even when saline was not infused. The high urine sodium excretion is a physiological response to the intensity of ADH action, as evident from an experiment by Leaf et al. who showed that continuous infusion of AVP increased the urine osmolality to 1,000 mOsm/kg, followed by high urine sodium excretion of 100–200 mmol/day (24). The natriuresis allows the excess extracellular fluid to be diuresed (4).

Hyponatremia affects up to 60% of patients with subarachnoid hemorrhage (25) and 13% of patients with TBI (26), with the major causes being SIADH or CSW (27, 28). Studies showed conflicting results on whether CSW or SIADH is the predominant cause of hyponatremia in such brain injuries, with reports of SIADH accounting for >80% of hyponatremia in patients post-TBI (10), and another reporting CSW in 0.8–34.6% in TBI patients (29). As the biochemistry results of SIADH and CSW are similar apart from much higher urine sodium concentration expected in the latter, the main differentiating factor is that of volume status, where CSW is associated with hypovolemia (5). However, volume status can be difficult to accurately determine (30). This is also described in a case reported by Chua et al. where the patient's hyponatremia worsened with intravenous fluids given for presumed CSW, leading to an eventual diagnosis of SIADH (16). Similarly in our first case, the patient's hyponatremia worsened following intravenous fluids (drop in serum sodium from 137 to 125 mmol/L). In a hypovolemic state, volume expansion would usually result in the loss of stimulus for vasopressin secretion and promote water diuresis with improvement in serum sodium level (11), although this may be insufficient to normalize hyponatremia in a severe salt wasting state such as in severe CSW. The clinical conundrum of the cause of hyponatremia in patients with brain pathologies (SIADH vs. CSW) remains in clinical practice. The importance of distinguishing the above has been questioned, since both conditions cause negative sodium balance and hypertonic saline can be used in both cases (30). However, the clinical distinction is important because the management differs significantly (4, 8, 9, 28).

Urine sodium levels in SIADH have been described to average 70–80 mmol/L (31, 32). Levels exceeding 100–150 mmol/L are typically seen in CSW, where Arieff et al. described urine sodium levels of 153 ± 50 mmol/L in CSW vs. 75 ± 41 mmol/L in post-operative patients with SIADH (31). Thus, the authors proposed that CSW can be differentiated from SIADH based on the urine sodium concentration. However, from our literature review, we identified several case reports of moderate-severe SIADH with urine sodium levels >100 mmol/L (Table 1) (13–20). This illustrated that although SIADH is typically associated with urine sodium levels of 30–100 mmol/L, there are several cases where urine sodium levels exceeded 100 mmol/L, with two cases even exceeding 200 mmol/L (16, 20). Similarly, our first case (post-TBI SIADH) also had a markedly raised urine sodium level of 204 mmol/L. Thus, the degree of elevation of urine sodium concentration alone cannot reliably differentiate between SIADH and CSW. In some cases of TBI, it remains unclear whether SIADH or CSW predominates in the etiology of hyponatremia and have been postulated to co-exist by Shen et al. (33) who reported 4 cases of refractory hyponatremia with extremely high 24-hour urine sodium excretion (700–1200 mmol/day). A triple regimen of IV fluids, hydrocortisone, and furosemide was prescribed.

It is unclear why some individuals have a predisposition to recurrent SIADH which can be severe, even to benign pathologies or commonly used medications such as proton-pump inhibitors, or to pain (4, 9). To our knowledge, there have not been any reported cases of SIADH precipitated by gastritis. Yet, such a scenario was observed in case 2 who required ICU admission previously for hyponatremia-induced altered mental status, with the next episode of SIADH 4 years later. It is likely that the water intake of 2–3L/day caused further dilutional hyponatremia in the setting of marked ADH activation, as reflected by the high urine osmolality and urine sodium concentration. While the fall in serum osmolality would have usually suppressed thirst, the patient continued to drink as self-remedy for his abdominal symptoms, thus resulting in a vicious cycle of worsening hyponatremia (34).

Predictors of failure of fluid restriction in the treatment of SIADH include urine osmolality >500 mOsm/kg and serum sodium concentration rise by <2 mmol/L/day in 24–48 h on a fluid restriction of 1L/day (4, 12). A urine/plasma sodium and potassium electrolyte ratio >1.0 indicates that fluid restriction alone, even to minimal amounts, would unlikely be sufficient to normalize serum sodium level (35). In a study of 106 patients with SIADH, the authors showed that urine sodium concentrations of ≥130 mmol/L compared to urine osmolality ≥500 mOsm/kg had higher specificity but equal sensitivity to predict failure to respond to fluid restriction, independent of diuretic use (12). This study showed that high levels of urine sodium and urine osmolality were individually associated with non-response to fluid restriction [odds ratio (OR) 15.0, 95% confidence interval (CI) 2.4–95.8, *P* = 0.004 and OR 34.8, 95% CI 1.2–1038.8, *P* = 0.041, respectively] (12). A randomized controlled trial studying 46 patients with SIADH and their response to fluid restriction found that baseline urine osmolality or urine/plasma electrolyte ratio did not predict treatment response to fluid restriction, however the study was underpowered (23). In another study of 84 cases of SIADH after head injury, the severe SIADH group compared with the mild SIADH group had higher urine osmolality and 24-h urine sodium concentrations (41–250 vs. 32–106 mmol/L) (36). Apart from these studies, our literature review of case reports of hyponatremia and high urine sodium concentration revealed that most of these patients eventually required pharmacological therapy on top of fluid restriction (hypertonic saline, tolvaptan, demeclocycline), Table 1 (14, 15, 17, 18). The presence of predictive factors (increase in serum sodium concentration <2 mmol/L/day in 24–48 h on fluid restriction, urine osmolality >500 mOsm/kg and high urine sodium concentration >130 mmol/L) in case 1 indicated that further pharmacological measures were likely required for successful management of SIADH. Instead, in case 2, the rapid reduction of urine sodium from 187 to 50 mmol/L the next day suggested that fluid restriction was likely to be successful.

While vaptan therapy would be effective in treating SIADH, its use in CSW is contraindicated due to the risk of hypovolemia. Mineralocorticoid administration using fludrocortisone has been found to be useful and safe in small studies and case reports for CSW as well as SIADH (26, 37). CSW is mediated by atrial natriuretic peptide (ANP) and brain natriuretic peptide (BNP) which are released after cerebral trauma, which then inhibit the release of aldosterone from the adrenal glands (38). ANP and BNP levels are usually normal in SIADH, but have also been described to be elevated in some case series (39). Fludrocortisone acts on the distal tubules in kidney to promote reabsorption of water and sodium, successfully restoring fluid volume status. Thus, it has been suggested to be given as a trial of treatment (40, 41). We found that fludrocortisone given in low doses (0.05 mg once a day) over a short period was helpful in case 1 to gradually normalize the serum sodium concentration.

## Conclusion

In conclusion, markedly raised urine sodium concentrations can be seen in SIADH, particularly in cases that are severe and refractory to fluid restriction. More studies are needed to determine the threshold of high urine sodium concentration with urine osmolality to predict severity, outcomes, and indication for early pharmacological therapy.

## Data Availability Statement

The original contributions presented in the study are included in the article/supplementary material, further inquiries can be directed to the corresponding author.

## Ethics Statement

Written informed consent was obtained from the individual(s) for the publication of any potentially identifiable images or data included in this article.

## Author Contributions

LL, ST, and WL: patient diagnosis and management. LL and ST: data collection and draft manuscript preparation. WL: revising manuscript critically. All authors reviewed and approved the final version of the manuscript.

## Conflict of Interest

The authors declare that the research was conducted in the absence of any commercial or financial relationships that could be construed as a potential conflict of interest.

## Publisher's Note

All claims expressed in this article are solely those of the authors and do not necessarily represent those of their affiliated organizations, or those of the publisher, the editors and the reviewers. Any product that may be evaluated in this article, or claim that may be made by its manufacturer, is not guaranteed or endorsed by the publisher.
